# Comparative Mortality of Five Large Cities for September

**Published:** 1869-11

**Authors:** Henry S. Chase


COMPARATIVE MORTALITY OF FIVE LARGE CITIES FOR SEPTEMBER.—We take the following from the St. Louis *Democrat.* Notwithstanding the palpable errors in the estimate of the population of some of the cities named, the statement is of interest. Giving to Cincinnati the population to which she is properly entitled in the estimate, it will be seen that her death rate is far below that of either of the other cities mentioned:

I have obtained from the boards of health of the following cities the mortality for the month of September, 1869:

| | | | | |
|---|---|---|---|---|
| New Orleans, | population, | 200,000, | deaths | 461. |
| Cincinnati, | " | 230,000, | " | 298. |
| Boston | " | 240,000, | " | 497. |
| St. Louis, | " | 240,000, | " | 646. |
| Chicago, | " | 252,000, | " | 814. |

| | | | | | |
|---|---|---|---|---|---|
| One person | died | of every | 309 | inhabitants | in Chicago. |
| " | " | " | 371 | " | in St. Louis. |
| " | " | " | 484 | " | in Boston. |
| " | " | " | 773 | " | in Cincinnati. |
| " | " | " | 433 | " | in New Orleans. |

HENRY S. CHASE, D.D.S.

